# RELION: Implementation of a Bayesian approach to cryo-EM structure determination

**DOI:** 10.1016/j.jsb.2012.09.006

**Published:** 2012-12

**Authors:** Sjors H.W. Scheres

**Affiliations:** MRC Laboratory of Molecular Biology, Hills Road, Cambridge CB2 0QH, UK

**Keywords:** Electron microscopy, Single-particle analysis, Maximum likelihood, Image processing, Software development

## Abstract

RELION, for REgularized LIkelihood OptimizatioN, is an open-source computer program for the refinement of macromolecular structures by single-particle analysis of electron cryo-microscopy (cryo-EM) data. Whereas alternative approaches often rely on user expertise for the tuning of parameters, RELION uses a Bayesian approach to infer parameters of a statistical model from the data. This paper describes developments that reduce the computational costs of the underlying maximum a posteriori (MAP) algorithm, as well as statistical considerations that yield new insights into the accuracy with which the relative orientations of individual particles may be determined. A so-called gold-standard Fourier shell correlation (FSC) procedure to prevent overfitting is also described. The resulting implementation yields high-quality reconstructions and reliable resolution estimates with minimal user intervention and at acceptable computational costs.

## 1 Introduction

Macro-molecular structure determination by single-particle analysis of electron cryo-microscopy (cryo-EM) images is a rapidly evolving field. Over the past two decades many reconstructions that reveal secondary structure elements have been obtained, e.g. see (Boettcher et al., 1997; Lau and Rubinstein, 2012; Lander et al., 2012), and recently several reconstructions to near-atomic resolution have been reported (Wolf et al., 2010; Liu et al., 2010; Yang et al., 2012). Improvements in electron microscopes and better computational tools for image processing have been important contributors to these successes. Moreover, on-going hardware developments such as direct-electron detectors (Milazzo et al., 2011; Brilot et al., 2012; Bammes et al., 2012) and phase-plates (Nagayama, 2011; Barton et al., 2011; Fukuda et al., 2012) are expected to improve data quality even further in the near future. This is likely to enhance the applicability of cryo-EM structure determination, as less noisy images will allow the visualization of smaller macro-molecular complexes.

The increased applicability of the technique is expected to attract new researchers to the field. Because conventional data collection and processing procedures often rely on user expertise, the needs for improved ease-of-use and automation are now widely recognized. More convenient data collection schemes are being developed through a combination of automated data acquisition software (Suloway et al., 2005) and improvements in the latest generation electron microscopes (Shrum et al., in press; Fischer et al., 2010). To cope with the large amounts of data from these experiments, semi-automated image processing pipelines and dedicated electronic notebooks have been proposed (Lander et al., 2009; Ludtke et al., 2003). Continuing developments in these areas are expected to increase the accessibility of cryo-EM structure determination to inexperienced users.

However, many cryo-EM projects still suffer from important hurdles in image processing that cannot be overcome by automation and increased volumes of data alone. Existing image processing procedures often comprise a concatenation of multiple steps, such as particle alignment, class averaging, reconstruction, resolution estimation and filtering. Many of these steps involve the tuning of specific parameters. Whereas appropriate use of these procedures may yield useful results, suboptimal parameter settings or inadequate combinations of the separate steps may also lead to grossly incorrect structures, thus representing a potential pitfall for newcomers to the field.

Recently, I described a Bayesian approach to cryo-EM structure determination, in which the reconstruction problem is expressed as the optimization of a single target function (Scheres, 2012). In particular, the reconstruction problem is formulated as finding the model that has the highest probability of being the correct one in the light of both the observed data and available prior information. Optimization of this posterior distribution is called maximum a posteriori (MAP), or regularized likelihood optimization. The Bayesian interpretation places the cryo-EM structure determination process on a firm theoretical basis, where explicit statistical assumptions about the model and the data, as well as the optimization strategy itself, can be discussed and improved if deemed necessary. Whereas conventional refinement procedures employ many *ad hoc* parameters that need to be tuned by an expert user, the Bayesian approach iteratively learns most parameters of the statistical model from the data themselves.

This paper describes the implementation of the Bayesian approach to single-particle reconstruction in the stand-alone computer program RELION, which stands for REgularized LIkelihood OptimizatioN. The theoretical implications of the statistical approach represent a huge challenge for its implementation in a useful computer program. Various algorithmic developments are described that allow MAP optimization of single-particle reconstructions at an acceptable computational cost. Moreover, the theoretical framework provided by the Bayesian approach may yield valuable insights into outstanding questions. As an example of this, I will describe an approach that uses the statistical data model to estimate the accuracy with which individual particles may be aligned and to quantify the contribution of different frequencies to this. Finally, because in principle some degree of overfitting might still go by unnoticed in the previously proposed MAP optimization approach (Scheres, 2012), a new procedure is described that eradicates the possibility of overfitting by the use of so-called “gold-standard” FSC calculations (Henderson et al., 2012; Scheres et al., 2012). Application of RELION to both simulated and experimental data illustrates that reconstructions that are free from overfitting may be obtained in a highly objective manner, without compromising reconstruction quality and at acceptable computational costs.

## 2 Approach

### 2.1 Theoretical background

MAP refinement of cryo-EM single-particle reconstructions is based on the following linear model in Fourier space:

(1) $$X_{\mathrm{ij}}={\text{CTF}}_{\mathrm{ij}}\overset{L}{\underset{l=1}{\sum }}P_{\mathrm{jl}}^{\phi }V_{\mathrm{kl}}+N_{\mathrm{ij}}\text{,}$$

where:

• $X_{\mathrm{ij}}$ is the *j*th component, with j=1,…,J, of the 2D Fourier transform $X_{i}$ of the *i*th experimental image, with i=1,…,N.
• ${\text{CTF}}_{\mathrm{ij}}$ is the *j*th component of the contrast transfer function for the *i*th image.
• $V_{\mathrm{kl}}$ is the *l*th component, with l=1,…,L, of the 3D Fourier transform $V_{k}$ of the *k*th of *K* underlying structures in the data set. Multiple structures *K* may be used to describe structural heterogeneity in the data, and *K* is assumed to be known. All components $V_{\mathrm{kl}}$ are assumed to be independent, zero-mean, and Gaussian distributed with variance $\tau_{\mathrm{kl}}^{2}$.
• ${\text{P}}^{\phi }$ is a J×L matrix of elements $P_{\mathrm{jl}}^{\phi }$. The operation $\sum_{l=1}^{L}P_{\mathrm{jl}}^{\phi }V_{\mathrm{kl}}$ for all *j* extracts a slice out of the 3D Fourier transform of the *k*th underlying structure, and ϕ defines the orientation of the 2D Fourier transform with respect to the 3D structure, comprising a 3D rotation and a phase shift accounting for a 2D origin offset in the experimental image. Similarly, the operation $\sum_{j-1}^{J}{\text{P}}_{lj}^{\phi^{T}}{\text{X}}_{ij}$ for all *l* places the 2D Fourier transform of an experimental image back into the 3D transform.
• $N_{\mathrm{ij}}$ is noise in the complex plane, which is assumed to be independent, zero-mean, and Gaussian distributed with variance $\sigma_{\mathrm{ij}}^{2}$.

Imagining an ensemble of possible solutions, the reconstruction problem is formulated as finding the model with parameter set Θ that has the highest probability of being the correct one in the light of both the observed data X and the prior information Y. According to Bayes’ law, this so-called posterior distribution factorizes into two components:

(2) P(Θ|X,Y)∝P(X|Θ,Y)P(Θ|Y)

where the *likelihood* P(X|Θ,Y) quantifies the probability of observing the data given the model, and the *prior* P(Θ|Y) expresses how likely that model is given the prior information. The likelihood is computed based on the assumption of independent, zero-mean Gaussian noise in the images, and one marginalizes over the orientations ϕ and class assignments *k*. The variance $\sigma_{\mathrm{ij}}^{2}$ of the noise components is unknown and will be estimated from the data. Variation of $\sigma_{\mathrm{ij}}^{2}$ with resolution allows the description of non-white, or coloured noise. The prior is based on the assumption that the Fourier components of the signal are also independent, zero-mean and Gaussian distributed with unknown and resolution-dependent variance $\tau_{\mathrm{kl}}^{2}$ (see Scheres, 2012 for more details). The model $\overset{ˆ}{\Theta }$, including all $V_{\mathrm{kl}}\text{,}\sigma_{\mathrm{ij}}^{2}$ and $\tau_{\mathrm{kl}}^{2}$, that optimizes the posterior distribution P(Θ|X,Y) is called the *maximum a posteriori* (MAP) estimate. Note that previously discussed ML methods in the Fourier domain (Scheres et al., 2007b) aimed to optimize P(X|Θ,Y).

Optimisation of P(Θ|X,Y) may be achieved by the expectation–maximization algorithm (Dempster et al., 1977), in which case the following iterative algorithm is obtained:

(3) $$V_{\mathrm{kl}}^{\text{(n+1)}}=\frac{\sum_{i=1}^{N}\int_{\phi }\Gamma_{\mathrm{ik}\phi }^{\text{(n)}}\sum_{j=1}^{J}P_{\mathrm{lj}}^{\phi^{T}}\frac{{\text{CTF}}_{\mathrm{ij}}X_{\mathrm{ij}}}{{\sigma_{\mathrm{ij}}^{2}}^{\text{(n)}}}d\phi }{\sum_{i=1}^{N}\int_{\phi }\Gamma_{\mathrm{ik}\phi }^{\text{(n)}}\sum_{j=1}^{J}P_{\mathrm{lj}}^{\phi^{T}}\frac{{\text{CTF}}_{\mathrm{ij}}^{2}}{{\sigma_{\mathrm{ij}}^{2}}^{\text{(n)}}}d\phi +\frac{1}{{\tau_{\mathrm{kl}}^{2}}^{\text{(n)}}}}\text{,}$$

(4) $${\sigma_{\mathrm{ij}}^{2}}^{\text{(n+1)}}=\frac{1}{2}\overset{K}{\underset{k=1}{\sum }}\int_{\phi }\Gamma_{\mathrm{ik}\phi }^{\text{(n)}}{\left|X_{\mathrm{ij}}-{\text{CTF}}_{\mathrm{ij}}\overset{L}{\underset{l=1}{\sum }}P_{\mathrm{jl}}^{\phi }V_{\mathrm{kl}}^{\text{(n)}}\right|}^{2}d\phi \text{,}$$

(5) $${\tau_{\mathrm{kl}}^{2}}^{\text{(n+1)}}=\frac{1}{2}{\left|V_{\mathrm{kl}}^{\text{(n+1)}}\right|}^{2}\text{,}$$

where $\Gamma_{\mathrm{ik}\phi }^{\text{(n)}}$ is the posterior probability of class assignment *k* and orientation assignment ϕ for the *i*th image, given the model at iteration number (n). It is calculated as follows:

(6) $$\Gamma_{\mathrm{ik}\phi }^{\text{(n)}}=\frac{P(X_{i}|k\text{,}\phi \text{,}\Theta^{\text{(n)}}\text{,}Y)P(k\text{,}\phi |\Theta^{\text{(n)}}\text{,}Y)}{\sum_{k^{\prime }=1}^{K}\int_{\phi^{\prime }}P(X_{i}|k^{\prime }\text{,}\phi^{\prime }\text{,}\Theta^{\text{(n)}}\text{,}Y)P(k^{\prime }\text{,}\phi^{\prime }|\Theta^{\text{(n)}}\text{,}Y)d\phi^{\prime }}\text{,}$$

with:

(7) $$P(X_{i}|k\text{,}\phi \text{,}\Theta^{\text{(n)}}\text{,}Y)=\overset{J}{\underset{j=1}{\prod }}\frac{1}{2\pi {\sigma_{\mathrm{ij}}^{2}}^{\text{(n)}}}\exp\left(\frac{{\left|X_{\mathrm{ij}}-{\text{CTF}}_{\mathrm{ij}}\sum_{l=1}^{L}P_{\mathrm{jl}}^{\phi }V_{\mathrm{kl}}^{\text{(n)}}\right|}^{2}}{-2{\sigma_{\mathrm{ij}}^{2}}^{\text{(n)}}}\right)\text{,}$$

and $P(k\text{,}\phi |\Theta^{\text{(n)}}\text{,}Y)$ may be used to express prior information about the distribution of the hidden variables *k* and ϕ. In practice, the integrations over ϕ are replaced by (Riemann) summations over discretely sampled orientations, and translations are limited to a user-defined range. Also, the power of the signal, $\tau_{\mathrm{kl}}^{2}$, and of the noise, $\sigma_{\mathrm{ij}}^{2}$, are estimated as 1D vectors, varying only with the resolution of Fourier components *j* and *l*.

The iterative algorithm in Eqs. (3)–(7) is started from an initial estimate for $V_{k}$: the starting model. If K>1, multiple different starting models are obtained by random division of the data set in the first iteration. The user controls the number of models *K* that is to be refined simultaneously. Initial estimates for $\tau_{\mathrm{kl}}$ and $\sigma_{\mathrm{ij}}$ are calculated from the power spectra of the starting model and individual particles, respectively.

It is important to note that the algorithm outlined above is a local optimizer. Thereby, the outcome of the refinement depends on the suitability of the starting model, and grossly incorrect starting models may lead to suboptimal results. Typically, to reduce bias to a possibly incorrect starting model, one applies a strong low-pass filter to the starting model.

### 2.2 Increasing computational speed: fast Fourier-space interpolation

Eqs. (3)–(7) represent a daunting computational challenge. Within each iteration, for every experimental image one has to evaluate the posterior probability $\Gamma_{\mathrm{ik}\phi }^{\text{(n)}}$ for all possible ϕ and *k*, and each image has to be back-projected into the 3D map with the corresponding weight for all ϕ and all *k*. Previous ML implementations reduced computational costs by keeping a set of pre-calculated 2D reference projections on a relatively coarsely sampled orientational grid in memory (Scheres et al., 2007a,b). Moreover, summations over all experimental images, in-plane rotations and translations were performed in 2D, and the corresponding weighted sums were also stored in memory. The resulting quadratic scaling of computer memory usage with the angular sampling rate in practice meant that ML refinements could not be performed with angular sampling rates finer than 10°, which seriously limited attainable resolutions.

RELION implements a drastically different approach. Instead of storing many 2D images in computer memory, it calculates projection and back-projection operations on-the-fly. The main advantage of this approach is that memory requirements no longer increase with increasing angular sampling rates, apart from storing a larger $\Gamma_{\mathrm{ik}\phi }^{\text{(n)}}$ array. However, because the (back-) projection operations have to be performed for many experimental images and a large number of orientations, this approach requires fast calculation of the (back-) projection operations in order to be computationally feasible.

As mentioned above, the projection and back-projection operations involve taking 2D slices out of a 3D Fourier transform, and putting them back in. This requires some sort of interpolation because the 3D Cartesian grid on which $V_{k}$ is sampled does not generally coincide with the 2D Cartesian grid of $X_{i}$. To speed up the calculations inside RELION, the 3D Fourier transform $V_{k}$ is oversampled twice by zero-padding of the map in real-space, and projection operations are then performed using linear interpolation in Fourier space. The linear interpolation scheme makes matrices $P^{\phi }$ very sparse, so that the computational cost of the projection operations is minimized and the integrals over ϕ in Eqs. (3)–(7) may be evaluated within reasonable time. To reduce artifacts in the projections, a reverse gridding correction (with a ${\text{sinc}}^{2}$-function) is applied to the 3D map prior to calculation of the Fourier transform.

A similar, inverse procedure is followed for the back-projection operations, where 2D Fourier transforms $X_{i}$ are placed into an oversampled 3D transform using the transpose of matrix $P^{\phi }$. However, the summation over all back-projected images in the numerator of Eq. (3) then results in a severely non-uniformly sampled 3D transform. This transform must be properly weighted before the actual reconstruction is obtained by an inverse Fourier transform operation, since straightforward division by the weights in the denominator of Eq. (3) would lead to unsatisfactory results. For this purpose, RELION implements a modified version of an iterative gridding reconstruction algorithm that was previously proposed for medical magnetic resonance imaging (MRI) (Pipe and Menon, 1999) and positron emission tomography (PET) (Matej and Lewitt, 2001). This algorithm is described in more detail in Appendix A.

### 2.3 Increasing computational speed: adaptive expectation–maximization

With the computational cost of the (back-) projection operations reduced, the most costly operation in Eqs. (3)–(7) is the calculation of the l2-norm in Eq. (7), which has to be evaluated for all i,k and ϕ. In particular, the orientations ϕ span a large 5D domain, comprising 3 rotations and 2 translations. Several approaches have previously been proposed to accelerate these calculations through domain reduction (Scheres et al., 2005; Tagare et al., 2010). In the domain reduction strategy, the integration over the entire domain is replaced by an integration over a significantly smaller sub-domain. Because in practice the posterior distribution $\Gamma_{\mathrm{ik}\phi }^{\text{(n)}}$ is close to zero for many *k* and ϕ, this turns out to be an effective way to approximate the total integration at strongly reduced computational costs.

RELION implements a modified version of the adaptive expectation maximization algorithm that was proposed by Tagare et al. (2010). For each experimental image, in a first pass $\Gamma_{\mathrm{ik}\phi }^{\text{(n)}}$ is evaluated over the entire domain using a relatively coarsely sampled grid of ϕ. The array of all $\Gamma_{\mathrm{ik}\phi }^{\text{(n)}}$ is sorted, and a sub-domain of all *k* and ϕ is selected that corresponds to the highest values of $\Gamma_{\mathrm{ik}\phi }^{\text{(n)}}$ that sum to a significant fraction ξ, typically 99.9%, of the total probability mass on the coarse grid. Then, in a second pass, $\Gamma_{\mathrm{ik}\phi }^{\text{(n)}}$ is evaluated only over the selected sub-domain using a finer grid.

The adaptive algorithm requires two discrete sampling grids of the continuous orientations ϕ: a coarse one and a fine one. To avoid a bias towards certain orientations, both grids ought to be uniformly sampled over the entire domain. For computational efficiency it is also convenient if the sampling points on the coarse grid can be related at little computational cost to their neighbouring points on the fine grid. For the sampling of the 2D translations, both requirements are easily fulfilled using Cartesian grids in Euclidian space. However, for the 3D orientations, there is no known point set that achieves uniform sampling.

RELION parameterizes 3D orientations by three Euler angles, and approximates a uniform sampling of the first two Euler angles on the sphere using the HEALPix framework (Gorski et al., 2005). The HEALPix approach was originally proposed for the field of astronomy (where pixelized images of the sky are represented on a sphere), and it has two characteristics that are particularly useful for the adaptive expectation–maximization algorithm outlined above: (i) it yields a reasonable approximation to a uniform sampling of the sphere so that bias towards certain orientations may be minimized; and (ii) it generates discrete grids in a hierarchical way that allows fast calculation of neighbouring sampling points in grids with distinct angular sampling rates. In particular, each subsequent grid in the hierarchy contains four times more sampling points than the previous one, yielding an angular sampling rate that is approximately twice as high.

The implemented adaptive expectation maximization algorithm uses a given grid in the HEALPix hierarchy for the coarse sampling of the first two Euler angles, and the next one in the hierarchy for the fine sampling. In addition, it uses a two times finer, linear sampling of the third Euler angle and of both translations in the fine grid. Thereby, the fine grid will have $2^{5}=32$ times more sampling points than the coarse sampling grid. Consequently, the maximum speed-up of the adaptive approach will be close to 32 (i.e. if only one sampling point contributes to 99.9% of the probability mass on the coarse grid). In practice, the posterior distributions are typically relatively broad during the initial stages of refinement (where low-resolution models provide less information to distinguish different orientations), and these distributions become more “spiky” towards convergence. Therefore, more orientations will contribute significantly to the probability mass on the coarse grid during the first few iterations when speed-ups are typically less pronounced, while towards the end of the refinement speed-ups become much more important.

### 2.4 Increasing computational speed: local orientational searches

Another effective approach to domain reduction is to limit the integrations to those orientations in the vicinity of the optimal orientations from the previous iteration. This approach is used in many structure determination procedures, and it is sometimes referred to as performing local angular searches. This approach may provide large speed-ups, but its effect on the quality of the reconstruction depends strongly on the assumption that the optimal orientations from the previous iteration are close to the true orientations. Therefore, local angular searches with fine orientational samplings are most useful during the later stages of refinement, after exhaustive searches with coarser samplings have provided orientations that are relatively close to the correct ones.

Inside the statistical framework, local angular searches may be implemented as a prior on the hidden variables. By setting $P(k\text{,}\phi |\Theta^{\text{(n)}}\text{,}Y)=0$ for orientations that are far away from the optimal ones in the previous iteration, integrations over those orientations may be avoided. Conventional local angular searches, where equal probabilities are given to orientations in a user-defined search range correspond to using a rectangular function for $P(k\text{,}\phi |\Theta^{\text{(n)}}\text{,}Y)$. RELION uses a truncated Gaussian function for $P(k\text{,}\phi |\Theta^{\text{(n)}}\text{,}Y)$, and integrations are limited to orientations within three times a user-defined standard deviation. This procedure downweights orientations that are relatively far away from the optimal orientations in the previous iterations, thereby providing a more continuous transition from orientations that are close to the previous ones and orientations that fall outside the user-defined search range.

### 2.5 Assessing alignment accuracy based on SNR considerations

The accuracy with which individual particles may be aligned remains an unknown in many structure determination procedures. However, this value is of great interest, as it may be used to predict the attainable resolution for a given data set. The effect of orientational errors may be modelled by a *B*-factor on the reconstruction, so that orientational errors of a given magnitude will limit the resolution in a predictable manner, e.g. see Table 2 in Henderson et al. (2011).

The statistical assumptions of the MAP approach may be used to estimate the accuracy with which orientational assignments can be made for a given model. If orientation $\phi^{\text{T}}$ is the true one for the *i*th image, then the ratio $R_{F/T}$ of the posterior probabilities of assigning a false orientation $\phi^{\text{F}}$ and the true orientation $\phi^{\text{T}}$ (for a given class assignment *k*, and assuming equal prior probabilities for both orientations) is given by:

(8) $$R_{\text{F}/\text{T}}=\frac{\Gamma_{\mathrm{ik}\phi^{\text{F}}}}{\Gamma_{\mathrm{ik}\phi^{\text{T}}}}=\exp\left(\overset{J}{\underset{j=1}{\sum }}\frac{{\left|{\text{CTF}}_{\mathrm{ij}}\sum_{l=1}^{L}P_{\mathrm{jl}}^{\phi^{\text{F}}}V_{\mathrm{kl}}-{\text{CTF}}_{\mathrm{ij}}\sum_{l=1}^{L}P_{\mathrm{jl}}^{\phi^{\text{T}}}V_{\mathrm{kl}}\right|}^{2}}{-2\sigma_{\mathrm{ij}}^{2}}\right)\text{.}$$

If $R_{\text{F}/\text{T}}$ is close to one for two neighbouring orientations, then these orientations cannot be distinguished from each other. On the other hand, if $R_{\text{F}/\text{T}}$ is very low, then the posterior probability of assigning the correct orientation is much larger than assigning the incorrect one, so that the correct orientation can readily be identified. Inside RELION, at every iteration one assumes for a random subset of 100 experimental images that the most likely orientations from the previous iteration are the correct ones, and one then modifies for each image each of the three Euler angles and two translations in small steps until $R_{\text{F}/\text{T}}<0.01$. The average values for the corresponding rotational and translational differences are reported by the program, and these values are considered to represent the accuracy with which different orientations may be distinguished reliably.

### 2.6 Preventing overfitting: “gold-standard” FSC calculations

In many structure determination procedures the resolution is assessed by FSC curves between reconstructions from halves of the data set, while a single model is used for the angular assignments. It is well-known that bias towards noise in this single model may lead to spurious correlations between the half-reconstructions. Over-optimistic low-pass filtering based on the inflated resolution estimates may then lead to further enhancement of the noise in the model. As a result, during multiple refinement iterations the amount of noise may gradually increase and final resolution estimates may be grossly exaggerated. This phenomenon has been called over-refinement, or overfitting. More realistic estimates of resolution may be obtained by refining a separate model for two independent halves of the data, so that FSC curves between the two half-reconstructions are free from spurious correlations. Such FSCs between independent reconstructions have been termed “gold-standard” FSCs (Henderson et al., 2012). As shown previously, “gold-standard” FSCs may be used to prevent overfitting without loss of reconstruction quality (Scheres et al., 2012).

Although MAP optimization was shown to effectively reduce overfitting, in theory some overfitting may still occur within the original MAP approach. If somehow noise would build up in the single reconstruction that is used for refinement, then the estimated power of the signal, through Eq. (5), would be inflated, which could then lead to overfitting. Although overfitting was observed to be much reduced compared to conventional refinement procedures, indications of a limited extent of overfitting in the MAP approach were indeed observed for very noisy data, see (Scheres, 2012) for more details.

To completely eradicate overfitting from the refinement process, an approach to estimate the power of the signal based on “gold-standard” FSC calculations was implemented inside the framework of MAP optimization. For this purpose, the data set is divided into two random halves at the outset of refinement, and two sets of model parameters Θ are refined separately, one for each half of the data. Because refinements with K>1 of independent random halves of the data might converge to distinct classification solutions, this procedure was only implemented for the K=1 case, and in the following all subscripts *k* have been dropped. At the end of every iteration, an FSC curve between the two independent reconstructions is calculated, and this curve is converted into an estimate for the resolution-dependent signal-to-noise ratio using:

(9) $${\text{SNR}}^{\text{MAP}}(\nu )=\frac{\text{FSC}(\nu )}{1-\text{FSC}(\nu )}\text{,}$$

which is then used to estimate the power spectrum of the underlying signal:

(10) $$\tau^{2}{\left(v\right)}^{\left(n\right)}=\frac{{\text{SNR}}^{\text{MAP}}(v)}{\frac{1}{N_{v}}\sum_{l\in v}^{N_{v}}\sum_{i=1}^{N}\int_{\phi }\Gamma_{i\phi }^{\left(n\right)}\sum_{j=1}^{J}{\text{P}}_{li}^{\phi^{T}}\frac{CTF_{ij}^{2}}{\sigma_{ij}^{2(n)}}d\phi }\text{,}$$

where l∈ν is used to indicate that the *l*th 3D Fourier component lies within resolution shell ν, and $N_{\nu }$ is the total number of Fourier components that lie within that resolution shell.

The estimated values for $\tau^{2}(\nu )$ are then used to calculate the optimal 3D linear filter for both reconstructions according to Eq. (3). Note that despite the 1D-character of $\tau^{2}(\nu )$ and FSC(ν), the modelled SNR in the Fourier domain may still be anisotropic through anisotropic CTF models and uneven orientational distributions in Eq. (3). Also note that Eq. (10) replaces Eq. (5) in the original MAP algorithm.

Only upon convergence of the refinement may the two subsets be joined to calculate a single reconstruction from all images. This final reconstruction will have a higher SNR than the two reconstructions from the independent halves of the data, but in order to prevent overfitting it may no longer be used in refinement. As suggested by Rosenthal and Henderson (2003), upon convergence, the FSC curve is modified as ${\text{FSC}}^{\prime }=\sqrt{2\text{FSC}(\nu )/(1+\text{FSC}(\nu ))}$ to estimate the resolution of the combined reconstruction. Consequently, the frequency where the gold-standard FSC curve passes through 0.143 indicates the estimated resolution of the map. Recent insights that take into account that the volume occupied by the particle is typically only a fraction of the entire reconstructed volume (Sindelar and Grigorieff, in press) may be considered in future versions of the program.

### 2.7 General implementation details

RELION is implemented as a stand-alone program, and its open-source C++ code is available for download from http://www2.mrc-lmb.cam.ac.uk/relion. All developments described above have been implemented in version 1.1. Pieces of code, e.g. for dealing with symmetry, Euler angle operations and image I/O, were copied and/or adapted from the open-source packages XMIPP (Sorzano et al., 2004) and BSOFT (Heymann and Belnap, 2007), and all Euler angle and symmetry conventions are in accordance with the 3D-EM standard conventions (Heymann et al., 2005). A graphical interface is provided to facilitate its use by novice users.

Following the strategy employed in BSOFT, all metadata I/O is through plain text files in the STAR format (Hall, 1991). This format provides a convenient way to store tables of label-value pairs in a highly structured manner that is similar to XML but much easier to read by humans. The crystallographic community makes extensive use of the STAR format through crystallographic information files (CIF) (Hall et al., 1991). The structured metadata I/O in RELION was designed to facilitate its incorporation into umbrella-like packages that provide a uniform interface to a range of other programs. Efforts to do so in APPION (Lander et al., 2009) and EMAN2 (Tang et al., 2007) are currently ongoing (personal communication with Bridget Carragher and Steven Ludtke, respectively).

Despite the above-mentioned algorithmic efforts to speed up calculations, RELION may still require considerable amounts of CPU depending on the task at hand. To further reduce computation times, RELION adopts a hybrid parallelization scheme at two distinct levels. Distributed-memory parallelization through the message passing interface (MPI) is employed to divide the data set into subsets of images that are processed in parallel. A work-on-demand implementation, where a master node dispatches relatively small jobs to slave nodes that request work whenever they are idle, allows an efficient use of heterogeneous computer clusters. Also the processing of the random halves of the data for the gold-standard FSC calculations is handled by MPI, where each half of the data is sent to a different subset of the slaves. At a lower level, shared-memory parallelization through POSIX threads is employed to further divide the work load of the MPI nodes. Each thread processes a subset of all orientations for each individual image. The distinct advantage of using threads over MPI is that all threads can access the same computer memory, so that the total amount of memory in modern multi-core computing nodes may be used more efficiently. Taken together, the hybrid parallelization approach provides maximum flexibility: both in terms of scalability and memory usage.

## 3 Experimental procedures

The procedures outlined above were evaluated using simulated as well as experimental data. First, a simulated density map, or phantom, was used to assess the accuracy and speed of the projection and back-projection operations. For this purpose, a set of atomic coordinates of the 70S ribosome (PDB-IDs 2J00 and 2J01) (Selmer et al., 2006) was converted to a density map of 128×128×128 voxels, with a voxel size of 2.8 Å, using the xmipp_convert_pdb2vol program (Sorzano et al., 2004). This map was projected in 5000 different orientations that were taken from a previously reported cryo-EM study on 70S ribosomes (Scheres et al., 2007a). The resulting projections were then back-projected in their perfect orientations to generate a reconstructed density map, and the accuracy of this projection/reconstruction cycle was assessed by FSC-curves between this reconstruction and the original phantom.

Second, general refinement behaviour and computational costs of the MAP optimization approach were tested using an experimental cryo-EM data set of 5168 GroEL particles that is distributed as part of a workshop on the EMAN2 software package (Tang et al., 2007). Using standard procedures in XMIPP, see (Scheres, 2010) for details, all particles were normalized, 115 particles were discarded after initial sorting, and the remaining 5053 particles were windowed to images of 128 × 128 pixels, with a pixel size of 2.12 Å. Refinements with these data were performed in symmetry group D7; a soft spherical mask with a diameter of 205 Å was applied to the reconstructions at every iteration; and the starting model was obtained from a 50 Å low-pass filtered GroEL map from a previous study (Scheres, 2012). Reconstruction quality was assessed by FSC calculations between the reconstructed maps and a symmetrized GroEL crystal structure (PDB-ID 1XCK) (Bartolucci et al., 2005) that was also used to assess GroEL reconstructions in a previous study (Scheres, 2012). All estimated $\tau^{2}$ values in these refinements were multiplied by a constant T=4. As explained in more detail in Scheres (2012), values of *T* in the range of 2–4 typically yield better maps than those obtained with the original algorithm.

Additional tests to assess alignment accuracies were performed using simulated data that were designed to be similar to the experimental GroEL data. The symmetrized GroEL crystal structure was converted to a density map, to which a *B*-factor of 350 Å^2^ and an arbitrary scale factor were applied to yield a phantom with a similar power spectrum as the reconstruction obtained from the experimental data. This phantom was then projected into 5053 orientations, which comprised small random perturbations of the optimal orientations as determined for the experimental particles. For each simulated particle, identical CTF parameters were used as estimated for the experimental particles, and independent Gaussian noise was added in the Fourier domain using the same power spectra as estimated for the experimental data. FSC curves with the original phantom were used to assess the quality of reconstructions from these images, while the known orientations of all particles allowed the calculation of histograms of orientational error distributions.

Finally, to further illustrate its general applicability, RELION was applied to three additional cryo-EM data sets: 50,330 β-galactosidase particles that were described by Scheres et al. (2012); 5403 hepatitis B capsids that were selected from re-scanned micrographs that were previously described by Boettcher et al. (1997); and 3700 recoated rotavirus particle (RP7) that were described by Chen et al. (2009). Crystal structures for these complexes are available: PDB-ID 3I3E for β-galactosidase (Dugdale et al., 2010); PDB-ID 1QGT for hepatitis B capsid (Wynne et al., 1999); and PDB-ID 1QHD for the rotavirus VP6 protein (Mathieu et al., 2001). FSC calculations of the reconstructed maps vs. these crystal structures were used to assess the quality of the refinement results.

All calculations described in this paper were performed on Dell M610 computing nodes of eight 2.4 GHz Xeon E5530 cores and 16 Gb of RAM each. Projection and back-projection operations with the phantom were performed using a single core, while all other calculations used the hybrid parallelization scheme to launch eight threads on each of seven nodes, i.e. using 56 cores in parallel.

## 4 Results and discussion

### 4.1 Accuracy of the Fourier-space interpolations

**Fig.1:**
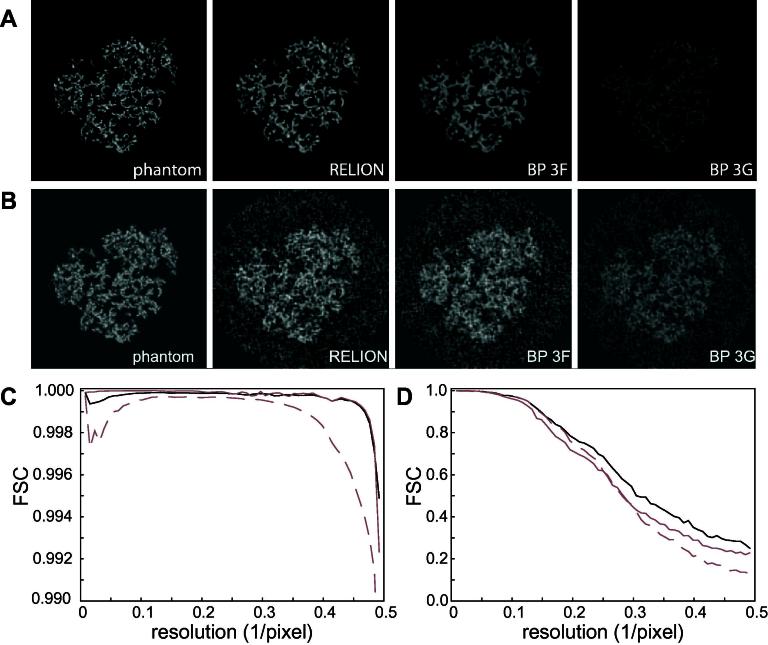
Accuracy of the projection/reconstruction cycle. (A) Central slices through the ribosome phantom and reconstructions made from 5000 noiseless projections in RELION and in SPIDER using commands BP3F or BP3G. All four images are on the same grey-scale. (B) As in A, but showing reconstructions made from 5000 noisy projections. (C) FSC curves between the phantom and reconstructions obtained from the noiseless projections in RELION (black), SPIDER BP3G (grey) and SPIDER BP3F (dashed grey). (D) As in C, but for reconstructions from the noisy projections. Note the difference in the *Y*-axis range between C and D.

The Fourier-space interpolation procedures outlined in Section 2.2 and Appendix A involve concessions to theory in order to obtain a computationally feasible approach. The accuracy of the resulting algorithms was assessed using a projection/reconstruction cycle with the ribosome phantom. In a first experiment, 5000 noiseless projections were generated and then back-projected again using RELION. The resulting reconstruction was compared with those obtained using two similar projection/reconstruction procedures in SPIDER (version 20.02) (Shaikh et al., 2008): one using a Kaiser–Bessel interpolation kernel (commands PR3Q and BP3F), the other using gridding (Penczek et al., 2004) (commands PR3G and BP3G), see Fig. 1A and C. All three approaches give FSC values higher than 0.99 up to the Nyquist frequency, although the Kaiser–Bessel interpolation kernel in SPIDER seems to perform slightly worse than the gridding approaches in SPIDER and RELION. The experiment was then repeated with projections in the same 5000 directions to which white Gaussian noise was added (with SNR = 0.1). In this case, the reconstruction obtained in RELION was somewhat better than both approaches in SPIDER (Fig. 1B and D).

Apparently, the interpolation scheme in RELION does not result in a deterioration of the reconstruction quality, although it is computationally highly efficient. RELION projection calculations took on average 0.9 ms and back-projections 1.2 ms. Accurate numbers were not estimated for the SPIDER calculations, as this would require modification of the source code. Yet, projections were generated in SPIDER every 50–100 ms, while back-projections took approximately 70–200 ms.

### 4.2 Acceleration of the MAP optimization algorithm

The efficiency of the remaining acceleration approaches was assessed using the cryo-EM dataset of 5053 GroEL particles. An initial refinement was performed with minimal acceleration. Following a pre-defined protocol of gradually increasing sampling rates, this calculation used exhaustive integrations over all rotations, and it did not use the adaptive expectation–maximization approach. The third column in Table 1 shows the wall-clock time required for these calculations. Given the relatively small size of the data set, the total required time of more than 24 days (while using 56 CPUs in parallel) was deemed excessive. The accelerating approaches that were outlined in Sections 2.3 and 2.4 were tested in two additional calculations. First, a similar run with adaptive expectation maximization was performed. Then, in addition to using adaptive expectation maximization, local angular searches were performed during iterations 21–40. For iterations 21–30, integrations were limited to within 5° from the orientations in the previous iteration (using a standard deviation of 1.66° for the Gaussian prior on the Euler angles). For iterations 31–40, orientational searches were limited to ±2.5° (using a standard deviation of 0.833°). Columns four and five in Table 1 show the required wall-clock times for these two runs. The adaptive expectation–maximization approach yields a speed-up that increases from 2-fold in the initial iterations to 24-fold in the final ones, while local angular searches provide an additional 8-fold acceleration during the last 10 iterations. FSC calculations indicated that all runs yielded a reconstruction that correlated up to 10 Å with the symmetrised crystal structure. The overall acceleration of more than two orders of magnitude between the run without acceleration and the run using both adaptive expectation maximization and local angular searches did not come at the cost of a noticeable deterioration of the reconstruction.

The procedure of gradually increasing sampling rates in itself represents an algorithmic approach to accelerate the MAP optimization. High sampling rates lead to accurate approximations of the continuous integrals in Eqs. (3)–(7), but come at considerable computational costs. However, too coarse angular samplings cannot represent the continuous integrals accurately, and may limit the resolution of the reconstruction. To test the efficiency of the procedure of gradually increasing sampling rates, an additional refinement was performed where the angular sampling rate was kept at a constant 1.8° for 40 iterations (using exhaustive integrations and adaptive expectation maximization). This run took 97 h of wall-clock time. Again a reconstruction was obtained that correlated up to 10 Å resolution with the symmetrised crystal structure. Apparently, using relatively coarse orientational samplings during the initial stages of refinement also yields a large increase in speed and does not have a noticable effect on the quality of the final reconstruction.

### 4.3 Assessment of angular assignment accuracy

**Fig.2:**
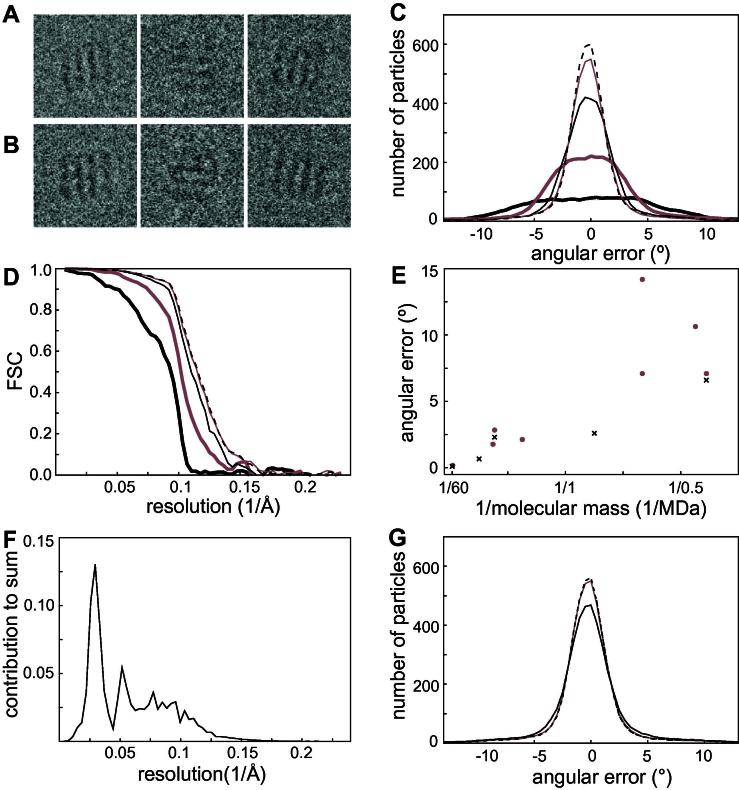
Assessment of angular accuracies. (A) Three simulated GroEL particles. (B) The experimental counterparts of the particles in A. (C) Distribution of the angular errors after a single iteration of refinement of a 10 Å low-pass filtered version of the phantom against the simulated data, using an angular sampling rate of 15° (bold black), 7.5° (bold grey), 3.7° (solid black), 1.8° (solid grey) or 0.9° (dashed black). (D) FSC with the phantom for the reconstructions from the refinements in C. (E) Experimentally determined angular accuracies based on tilt pair analysis (grey circles) compared to estimated angular accuracies based on the $R_{F/T}$ criterion (black crosses). The samples analyzed by tilt-pair analysis were rotavirus double-layered particle (50 MDa), chicken anemia virus (2.7 MDa), 70S ribosome (2.7 MDa), fatty acid synthase (2.6 MDa), pyruvate dehydrogenase (1.6 MDa), V and F-type ATPase (0.6 MDa), DNA-dependent protein kinase (0.47 MDa) and β-galactosidase (0.45 MDa). The specimens analyzed in RELION were rotavirus recoated particle (60 MDa), hepatitis B capsid (4 MDa), 70S ribosome (2.7 MDa), GroEL (0.8 MDa) and β-galactosidase (0.45 MDa). (F) Contribution of the different resolution shells to the summation inside the exponential in Eq. (8) for projections of the GroEL phantom with an angular distance of 2.7° between $\phi^{T}$ and $\phi^{F}$. (G) Angular error distributions after alignment of the simulated GroEL particles against the phantom map using an angular sampling of 1.8°. The maximum resolution used in the alignment was varied between 20 Å (solid black), 10 Å (solid grey) and Nyquist (dashed black).

As the correct orientations remain unknown in any reconstruction from experimental data, the estimation of the angular accuracy based on the $R_{\text{F}/\text{T}}$ criterion was first assessed using the simulated GroEL data set. Fig. 2A shows some simulated particles and Fig. 2B their experimental counterparts. RELION estimated an angular accuracy of 2.9° for the alignment of the simulated particles against a 10 Å low-pass filtered version of phantom. To evaluate the usefulness of this estimation, additional MAP optimizations were performed using a range of different angular sampling rates. In each calculation, a single iteration was performed with the filtered phantom map as a reference. The optimal orientations from these calculations, i.e. those orientations with the highest $\Gamma_{i\phi }$, were compared to the known orientations of the simulated particles. Fig. 2C shows the distributions of the resulting angular errors. As expected, the angular errors decrease with increasingly fine sampling rates from 15° to 1.8°. However, using angular sampling rates that are even finer only lead to minor further improvements, which is confirmed by FSC curves between a reconstruction that was made from the particles in their optimal orientations and the known phantom map (Fig. 2D). Using the finest tested angular sampling rate of 0.9°, the fraction of particles that had angular errors smaller than the estimated value of 2.9° for the first, second and third Euler angle were, 70%, 96% and 76%, respectively, illustrating the relevance of the estimated accuracy.

To further assess the relevance of the estimated angular assignment accuracies based on the $R_{\text{F}/\text{T}}$ criterion, the estimated values were also compared to experimentally accessible values as obtained by tilt-pair analysis. For a range of different specimens, Henderson et al. (2011) aligned pairs of images that were taken at different tilt-angles in the microscope against a model reconstruction. Based on the extent to which the two independently assigned orientations of each pair were compatible with the experimentally known tilt-axis transformation, the accuracy with which the (pairs of) orientations were assigned could be estimated. These values showed an expected trend of increasing angular assignment accuracy with increasing molecular weight of the specimen (grey circles in Fig. 2E). Note that the angular accuracies plotted are divided by $\sqrt{2}$ compared to the values given in Table 1 of Henderson et al. (2011) to take into account that the measurements concerned image pairs instead of individual images. As also discussed by those authors, the accuracy of the first image may actually be somewhat better because the second image of the tilt pair is affected by more radiation damage than the first one. Still, a very similar trend was observed for the angular assignment accuracies as estimated based on the $R_{\text{F}/\text{T}}$ criterion inside RELION for a different range of specimens (black crosses in Fig. 2E). The good overall agreement between the estimated values and the experimentally accessible values confirms the relevance of the $R_{\text{F}/\text{T}}$ criterion.

The SNR considerations that led to Eq. (8) may also provide useful insights into the relative contribution of different frequencies to the alignment of the individual particles. Based on the $R_{\text{F}/\text{T}}$ criterion, the accuracy with which the simulated GroEL particles may be aligned against the perfect phantom model was estimated to be 2.7°. Fig. 2F shows the average resolution-dependent contribution to the summation in Eq. (8) for a random subset of 100 particles and for orientations $\phi^{T}$ and $\phi^{F}$ that are 2.7° apart. The signal in cryo-EM images falls off much faster with resolution than the noise. Therefore, higher-resolution terms will typically contribute less than the lower resolution terms to the summation inside the exponential of Eq. (8), despite the fact that the number of Fourier components in the 2D images increases quadratically with resolution. In fact, more than half of the total sum is made up for by components up to 15 Å resolution, and components beyond 8 Å resolution contribute only marginally. The plot in Fig. 2F suggests that excluding frequencies below 10 Å resolution from the refinement would lead to worse orientational assignments and thus worse reconstructions, while including frequencies beyond 8 Å would hardly benefit reconstruction quality at all. To test these predictions, three additional alignments of the simulated GroEL particles against the phantom map were performed. In these calculations, the resolution of the data that were included in the alignment was limited to 20 Å, 10 Å and the Nyquist frequency, respectively. Fig. 2G shows the resulting angular error distributions for these calculations. As predicted, the angular assignments only improve slightly upon the inclusion of data in the range between 20 and 10 Å, and virtually no improvement is obtained by including even higher frequencies. These results are in excellent agreement with experimental observations that only low-medium resolution components contribute significantly to the alignment of individual images (Henderson et al., 2011).

### 4.4 The prevention of overfitting: “gold-standard” FSCs

As recognized previously, the observation that only the low-medium resolution components in individual particles have sufficiently high SNRs to contribute significantly to the alignment explains why overfitting may be prevented without loss of reconstruction quality using gold-standard FSCs (Scheres et al., 2012). At these resolutions, for most cryo-EM studies reconstructions from only half of the data are nearly indistinguishable from reconstructions from all data. Therefore, orientational assignments that use half-reconstructions as references are not expected to be worse than those based on a reconstruction from all data. As long as the two independent reconstructions are combined upon convergence, the resolution of this final reconstruction made from all particles is therefore not expected not be worse than the resolution obtained using a single model in refinement. Moreover, because overfitting is prevented the gold-standard FSC curve will be a better indicator of the true resolution of the map.

**Fig.3:**
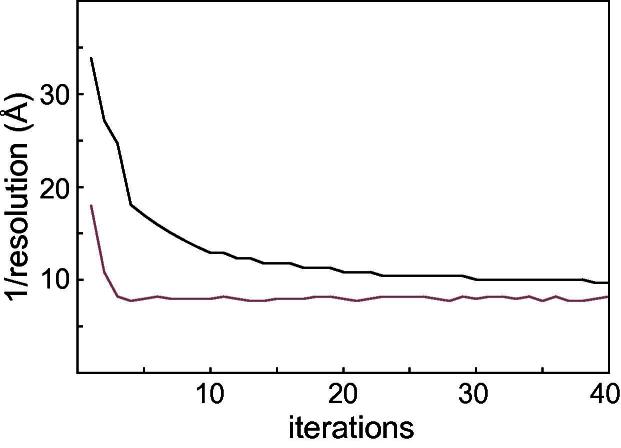
Reported resolutions for every iteration of a refinement using the original MAP algorithm (black) and a refinement using gold-standard FSCs (grey) to estimate signal strength.

To assess the use of gold-standard FSCs inside RELION, a run with the experimental GroEL particles that used Eqs. (9) and (10) was compared to a similar run using the original MAP algorithm. In both runs the angular sampling rate was fixed at 1.8° and exhaustive angular searches were performed using the adaptive expectation–maximization approach for 40 iterations. The reported resolutions for both runs at every iteration are shown in Fig. 3. The run based on gold-standard FSCs converges much faster than the original MAP approach. In the latter, the power of the reconstruction from the previous iteration is used to filter the reconstruction in the current iteration. This makes the expectation–maximization algorithm particularly slow to converge. The run using gold-standard FSCs to estimate signal strength converges faster because alignments based on the lower frequencies alone also yield correlations at higher frequencies. A reconstruction from all particles at the end of the run using gold-standard FSCs correlates up to 8.7 Å with the symmetrized GroEL crystal structure; the reconstruction from the original MAP approach up to 10 Å. It is also noteworthy that the multiplication of the estimated $\tau^{2}(\nu )$ values by the *ad hoc* constant T=4, which was observed to provide better convergence behaviour of the original MAP approach (Scheres, 2012), is no longer necessary in the gold-standard FSC approach.

### 4.5 3D auto-refine: a refinement procedure with minimal user intervention

Based on the results described above, a fully automated protocol was implemented for the refinement of structurally homogeneous data sets. The user only selects a relatively coarse initial orientational sampling, and this sampling rate is automatically increased during the refinement. For this purpose two convergence criteria are monitored: the estimated resolution (based on the gold-standard FSC curve) and the average changes in the optimal orientation and class assignments for all particles. Once both criteria no longer improve from one iteration to the next, the orientational sampling rates are increased. The rotational sampling is increased 2-fold by using the next Healpix grid. The translational sampling is adjusted to the estimated accuracy of the translational assignments based on the $R_{\text{F}/\text{T}}$ criterion. This process is repeated until the angular sampling employed is finer than the estimated angular accuracy as estimated using the $R_{\text{F}/\text{T}}$ criterion. During all iterations, the adaptive expectation–maximization algorithm is used, and from a user-defined angular sampling rate onwards, local angular searches are performed (within a search range of ±3 times the sampling rate). Upon convergence, a final iteration is performed where the two independent halves of the data are combined in a single reconstruction.

Apart from providing a starting model and a general description of the data, there are few parameters that need to be set by the user. The user decides on the frequency of an initial low-pass filter of the starting model, the user provides the diameter for a soft spherical mask to be applied to the reconstructions at every iteration, and the user sets the initial orientational sampling rates and the angular sampling rate from which to use local angular searches. The following rules of thumb may be of help to the inexperienced user. To reduce model bias, the filter on the starting model should be “as low-resolution as possible”. In most cases, a too low-resolution filter will result in a featureless blob that can no longer be refined. The recommended filter is somewhat higher than that. The diameter of the spherical mask should be choosen such that most of the solvent area is excluded, but care should be taken not to exclude any density of the particle. If the particle is far from spherical a user-defined mask may be provided (optional). This should preferably be a soft mask, with a continuous change from the solvent area (0-values in the mask) to the particle area (1-values in the mask). The optimal initial angular sampling rate and the angular sampling rate from which to use local angular searches mainly affects computational costs. Useful values for the initial angular sampling rate are 3.7° for icosahedral viruses, and 7.5° for lower-symmetry particles. The initial search range and step size of the translational sampling depends on the image and pixel size and on the accuracy with which the particles have been selected. Large values for the search range, combined with a small step size will considerably slow down the initial iterations. Often, searching ±6 pixels with a step size of 1 pixel is sufficient. Note that the centre of the translational searches for each particle is updated to the optimal translation in the previous iteration. Therefore, during more than one iteration the particles can still move over more pixels than the indicated search range.

**Fig.4:**
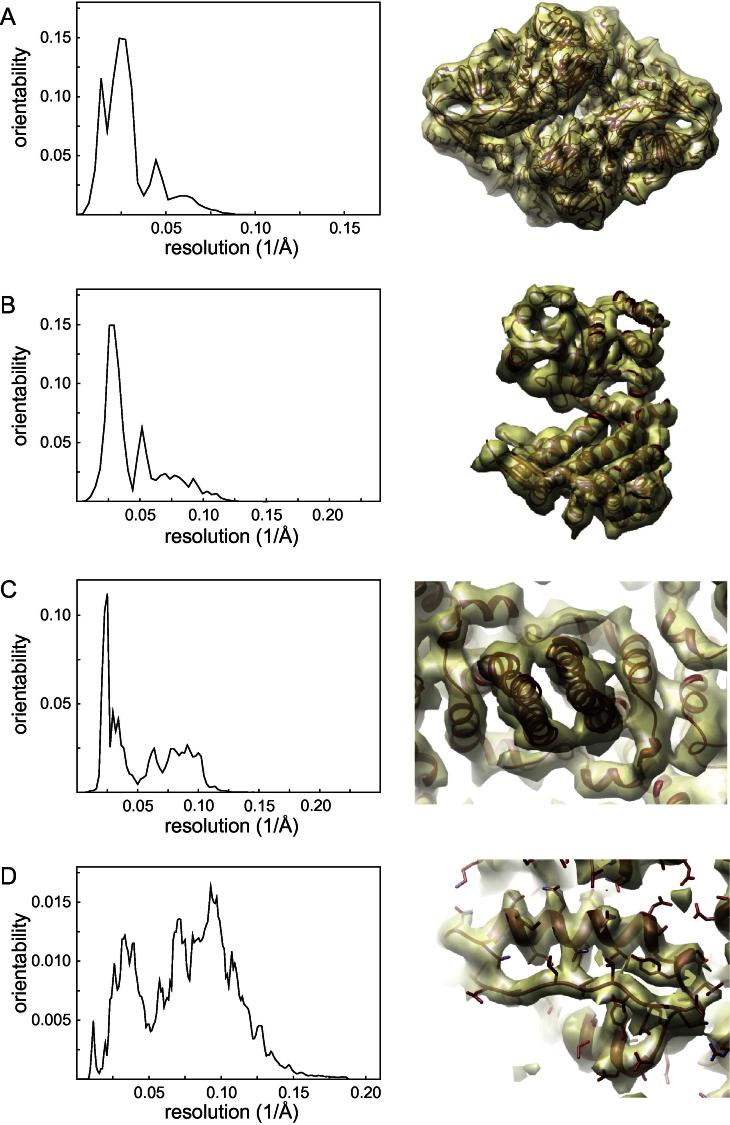
Results obtained with the *3D auto-refine* procedure for four cryo-EM data sets: (A) β-galactosidase, (B) GroEL, (C) hepatitis B capsid, and (D) recoated rotavirus. On the left are shown the resolution-dependent contributions to the orientability, i.e. to the sum inside the exponential in Eq. (8). On the right are shown representative pieces of reconstructed density (transparent yellow) with the corresponding fitted crystal structures inside (red). All maps were sharpened prior to visualization, using a *B*-factor of −1500, −750, −850 and −275 Å^2^ for the β-galactosidase, GroEL, hepatitis B capsid, and recoated rotavirus reconstructions, respectively.

To illustrate its versatility, the *3D auto-refine* procedure was applied to four cryo-EM data sets. Table 2 gives an overview of the data characteristics, the parameters used, and the resolutions obtained. Fig. 4 shows the resolution-dependent contributions to the orientability of the individual particles and representative parts of the reconstructed density maps. Objective indications of reconstruction quality were obtained by FSC calculations against available crystal structures. The resolution where these FSC curves dropped below 0.5 is reported in Table 2. Comparison of these values for the reconstruction obtained by RELION and previously reported reconstructions from the same data sets indicates that the elimination of user intervention from the 3D auto-refine procedure did not lead to a deterioration of the results. On the contrary, RELION yields reconstructions that are as good or better than those obtained by a variety of alternative refinement programs. The high-quality reconstructions come at a readily acceptable computational cost, the more so because the program does not need to be run multiple times in order to fine-tune *ad hoc* parameters.

## 5 Conclusions

Implementation of the procedures described here in the RELION program has resulted in a refinement tool that delivers state-of-the-art reconstructions at acceptable computational costs. The use of gold-standard FSCs to estimate resolution-dependent SNRs avoids overfitting and yields realistic resolution estimates (Scheres et al., 2012). Still, it is important to realize that RELION employs a local optimization algorithm (as most refinement programs do), which makes the outcome of the approach dependent on the quality of the starting model. Therefore, while the development of robust methods to generate *ab initio* starting models remains an active area of research, the development of better structure validation tools continues to be extremely relevant (Henderson et al., 2012).

In general, the Bayesian approach provides a statistical framework for the entire cryo-EM structure determination workflow. This framework was previously shown to provide new insights into the optimal filtering of 3D reconstructions (Scheres, 2012), and has now also been shown to be useful to predict the accuracy of alignment of individual particles and the relative contribution of the different frequencies therein. However, perhaps the greatest asset of the Bayesian approach is that most of its parameters are learned from the data themselves. Thereby, the careful tuning of *ad hoc* parameters by an expert user is avoided, which facilitates automation and increases the objectivity of cryo-EM structure determination.

## Figures and Tables

**Table 1:** Wall-clock times (in hours) for the given number of iterations in the first column for a run without acceleration (−/−); a run with adaptive expectation maximization (adap/−); and a run with both adaptive expectation maximization and local angular searches (adap/local).

Iter	Sampling (°)	−/−	adap/−	adap/local
1–10	7.5	0.8	0.4	ND
11–20	3.8	6.6	0.7	ND
21–30	1.8	56.3	3.1	2.7
31–40	0.9	535	21.6	2.8

**Table 2:** Refinement characteristics for four cryo-EM data sets.

	β-Galactosidase	groEL	Hepatitis B	Rotavirus
*Sample characteristics*				
Size (MDa)	0.45	0.8	4	60
Symmetry	D2	D7	I	I

*Microscopy settings*				
Microscope	FEI Polara G2	Jeol 3000SFF	Hitachi HF2000	FEI Tecnai F30
Voltage (kV)	80	300	200	300
Defocus range (μm)	1.2–2.7	1.9–3.2	1.0–2.0	1.2–2.9
Detector	Kodak SO163	Kodak SO163	Kodak SO163	Kodak SO163

*Data characteristics*				
Image size (pixel^2^)	100×100	128×128	220×220	400×400^a^
Pixel size (Å)	2.93	2.12	2.00	2.40
Number of particles	50,330	5053	5403	3700

*RELION parameters*				
Particle mask diameter (Å)	200	205	400	785
Initial low-pass filter (Å)	60	60	50	40
Initial angular sampling (°)	7.5	7.5	3.7	3.7
Local searches from (°)	1.8	1.8	0.5	0.5
Initial offset range (pixel)	6	6	6	6
Initial offset step (pixel)	1	1	1	1

*RELION results*				
Wall-clock time (h)	13.6	2.0	8.2	41.5
Reported resolution (Å)	9.8	8.2	7.3	5.6
Resolution vs. X-ray (Å)	10.1	8.4	7.3	4.4^b^

*Previous results*				
Refinement program	XMIPP^c^	EMAN2^d^	MRC	FREALIGN^e^
Reported resolution (Å)	13.9	8.4	7.4	≈6
Resolution vs. X-ray (Å)	12.7	8.7	7.5	4.4^b^

a The original rotavirus particles were downscaled by a factor of 2 to reduce memory requirements.

b After 13-fold non-icosahedral symmetry averaging, and for a masked region of the map comprising a single VP6 trimer.

c The results obtained using a gold-standard FSC version of the XMIPP projection matching protocol were used (Scheres et al., 2012).

d Results from the EMAN2 tutorial (version 2011) were downloaded from http://blake.bcm.edu/emanwiki/Ws2011/Eman2.

e FREALIGN results were downloaded from http://emlab.rose2.brandeis.edu/rota_recoated.

## Appendix A An iterative gridding reconstruction algorithm

A weighting function *W* (with values $W_{l}$ for all l=1,…,L) for the non-uniformly sampled transform in Eq. (3) is estimated in an iterative manner. Modifying Pipe and Menon (1999), the first iteration m=1 starts with:

(A.1) $$W^{\text{(m=1)}}=\frac{S}{(\Omega S)⊗\Psi }\text{,}$$

where *S* has values $S_{l}=1$ for all l=1,…,L grid points, ⊗ denotes convolution (evaluated at all grid points *l*), and the division is interpreted as a point-by-point division at all grid points. Ψ is a suitable interpolation function with a limited support in Fourier space. For this purpose, RELION uses a modified Kaiser–bessel function (or blob) of order m=0, radius r=1.9 and α=15. The term Ω, with $\Omega_{l}$ for all l=1,…,L grid points, is not present in Pipe and Menon (1999) and is set equal to the values in the denominator of Eq. (3), i.e.

(A.2) $$\Omega_{l}=\overset{N}{\underset{i=1}{\sum }}\int_{\phi }\Gamma_{\mathrm{ik}\phi }^{\text{(n)}}\overset{J}{\underset{j=1}{\sum }}P_{\mathrm{lj}}^{\phi^{T}}\frac{{\text{CTF}}_{\mathrm{ij}}^{2}}{{\sigma_{\mathrm{ij}}^{2}}^{\text{(n)}}}d\phi +\frac{1}{{\tau_{\mathrm{kl}}^{2}}^{\text{(n)}}}\;\text{for each}\;l\text{.}$$

The reason for this additional term lies in the observation that, because all 2D transforms $X_{i}$ were brought onto the same oversampled Cartesian grid, rather than processing each input data point separately (as in Pipe and Menon, 1999) it is much more efficient to evaluate the weighted sums over all images (in all orientations) at every grid point *l*. Also, because *W* is evaluated on a regular Cartesian grid, the convolution operation may be evaluated efficiently through Fourier transforms. It should however be noted that the linear interpolation that is used to bring all $X_{i}$ onto the oversampled 3D grid represents an arbitrary departure from the procedure proposed by Pipe and Menon, where speed considerations outweighted strict adherence to theory.

Again following Pipe and Menon, the weight is then iteratively refined using:

(A.3) $$W^{\text{(m+1)}}=\frac{W^{\text{(m)}}}{(\Omega W^{\text{(m)}})⊗\Psi }\text{.}$$

As pointed out by these authors as well, if during the initial iterations $W_{l}^{\text{(m)}}$ is regionally too large, e.g. because a preferred orientation has lead to a relatively densely sampled area in the transform, then the denominator in Eq. (A.3) will be greater than unity, reducing the next estimate $W_{l}^{\text{(m+1)}}$. Conversely, when $W_{l}^{\text{(m)}}$ is regionally too small, the next estimate will be increased. After several iterations (in RELION by default 10 iterations are performed), the values of $(\Omega W^{\text{(m)}})⊗\Psi$ are typically close to 1 for all *l*, so that $W^{\text{(m+1)}}\approx W^{\text{(m)}}$. Multiplication of the numerator of Eq. (3) with $W_{l}$ for all *l*, followed by an inverse Fourier transform then yields a real-space map, which is windowed to its original size and divided by the inverse transform of the interpolation kernel in Fourier space.

## References

[b0005] Bammes B.E., Rochat R.H., Jakana J., Chen D.-H., Chiu W. (2012). Direct electron detection yields cryo-EM reconstructions at resolutions beyond 3/4 Nyquist frequency. Journal of Structural Biology.

[b0010] Bartolucci C., Lamba D., Grazulis S., Manakova E., Heumann H. (2005). Crystal structure of wild-type chaperonin GroEL. Journal of Molecular Biology.

[b0015] Barton B., Rhinow D., Walter A., Schroeder R., Benner G. (2011). In-focus electron microscopy of frozen-hydrated biological samples with a Boersch phase plate. Ultramicroscopy.

[b0020] Brilot A.F., Chen J.Z., Cheng A., Pan J., Harrison S.C., Potter C.S., Carragher B., Henderson R., Grigorieff N. (2012). Beam-induced motion of vitrified specimen on holey carbon film. Journal of Structural Biology.

[b0025] Boettcher B., Wynne S.A., Crowther R.A. (1997). Determination of the fold of the core protein of hepatitis B virus by electron cryomicroscopy. Nature.

[b0030] Chen J.Z., Settembre E.C., Aoki S.T., Zhang X., Bellamy A.R. (2009). Molecular interactions in rotavirus assembly and uncoating seen by high-resolution cryo-EM. Proceedings of the National Academy of Sciences of the United States of America.

[b0035] Dempster A.P., Laird N.M., Rubin D.B. (1977). Maximum-likelihood from incomplete data via the EM algorithm. Journal of the Royal Statistical Society: Series B.

[b0040] Dugdale M.L., Dymianiw D.L., Minhas B.K., D’Angelo I., Huber R.E. (2010). Role of Met-542 as a guide for the conformational changes of Phe-601 that occur during the reaction of β-galactosidase (*Escherichia coli*).. Biochemistry and Cell Biology.

[b0045] Fischer N., Konevega A.L., Wintermeyer W., Rodnina M.V., Stark H. (2010). Ribosome dynamics and tRNA movement by time-resolved electron cryomicroscopy. Nature.

[b0050] Fukuda Y., Nagayama K. (2012). Zernike phase contrast cryo-electron tomography of whole mounted frozen cells. Journal of Structural Biology.

[b0055] Gorski K.M., Hivon E., Banday A.J., Wandelt B.D., Hansen F.K. (2005). HEALPix – a framework for high resolution discretization, and fast analysis of data distributed on the sphere. The Astrophysical Journal.

[b0060] Hall S.R. (1991). The STAR file: a new format for electronic data transfer and archiving. Journal of Chemical Information and Computer Sciences.

[b0065] Hall S.R., Allen F.H., Brown I.D. (1991). The crystallographic information file (CIF): a new standard archive file for crystallography. Acta Crystallographica Section A Foundations of Crystallography.

[b0070] Henderson R., Chen S., Chen J.Z., Grigorieff N., Passmore L.A. (2011). Tilt-pair analysis of images from a range of different specimens in single-particle electron cryomicroscopy. Journal of Molecular Biology.

[b0075] Henderson R., Sali A., Baker M.L., Carragher B., Devkota B. (2012). Outcome of the first electron microscopy validation task force meeting. Structure (London, England: 1993).

[b0080] Heymann J.B., Belnap D.M. (2007). BSOFT: image processing and molecular modeling for electron microscopy. Journal of Structural Biology.

[b0085] Heymann J.B., Chagoyen M., Belnap D.M. (2005). Common conventions for interchange and archiving of three-dimensional electron microscopy information in structural biology. Journal of Structural Biology.

[b0090] Lander G.C., Estrin E., Matyskiela M.E., Bashore C., Nogales E. (2012). Complete subunit architecture of the proteasome regulatory particle. Nature.

[b0095] Lander G.C., Stagg S.M., Voss N.R., Cheng A., Fellmann D. (2009). APPION: an integrated, database-driven pipeline to facilitate EM image processing. Journal of Structural Biology.

[b0100] Lau W.C.Y., Rubinstein J.L. (2012). Subnanometre-resolution structure of the intact *Thermus thermophilus* H+-driven ATP synthase. Nature.

[b0105] Liu H., Jin L., Koh S.B.S., Atanasov I., Schein S. (2010). Atomic structure of human adenovirus by cryo-EM reveals interactions among protein networks. Science (New York, NY).

[b0110] Ludtke S.J., Nason L., Tu H., Peng L., Chiu W. (2003). Object oriented database and electronic notebook for transmission electron microscopy. Microscopy and Microanalysis.

[b0115] Matej S., Lewitt R.M. (2001). 3D-FRP: direct fourier reconstruction with fourier reprojection for fully 3-d PET. IEEE Transactions on Nuclear Science.

[b0120] Mathieu M., Petitpas I., Navaza J., Lepault J., Kohli E. (2001). Atomic structure of the major capsid protein of rotavirus: implications for the architecture of the virion. The EMBO Journal.

[b0125] Milazzo A.-C., Cheng A., Moeller A., Lyumkis D., Jacovetty E. (2011). Initial evaluation of a direct detection device detector for single particle cryo-electron microscopy. Journal of Structural Biology.

[b0130] Nagayama K. (2011). Another 60 years in electron microscopy: development of phase-plate electron microscopy and biological applications. Journal of Electron Microscopy.

[b0135] Penczek P.A., Renka R., Schomberg H. (2004). Gridding-based direct fourier inversion of the three-dimensional ray transform. Journal of the Optical Society of America. A, Optics, Image Science, and Vision.

[b0140] Pipe J.G., Menon P. (1999). Sampling density compensation in MRI: rationale and an iterative numerical solution. Magnetic Resonance in Medicine: Official Journal of the Society of Magnetic Resonance in Medicine/Society of Magnetic Resonance in Medicine.

[b0145] Rosenthal P.B., Henderson R. (2003). Optimal determination of particle orientation, absolute hand, and contrast loss in single-particle electron cryomicroscopy. Journal of Molecular Biology.

[b0150] Scheres S.H.W. (2010). Classification of structural heterogeneity by maximum-likelihood methods. Cryo-EM, Part B: 3-D Reconstruction.

[b0155] Scheres S.H.W. (2012). A bayesian view on cryo-EM structure determination. Journal of Molecular Biology.

[b0160] Scheres S.H.W., Chen S. (2012). Prevention of overfitting in cryo-EM structure determination. Nature Methods.

[b0165] Scheres S.H.W., Gao H., Valle M., Herman G.T., Eggermont P.P.B. (2007). Disentangling conformational states of macromolecules in 3D-EM through likelihood optimization. Nature Methods.

[b0170] Scheres S.H.W., Nunez-Ramirez R., Gomez-Llorente Y., San Martin C., Eggermont P.P.B. (2007). Modeling experimental image formation for likelihood-based classification of electron microscopy data. Structure.

[b0175] Scheres S.H.W., Valle M., Carazo J.M. (2005). Fast maximum-likelihood refinement of electron microscopy images. Bioinformatics.

[b0180] Selmer M., Dunham C.M., Murphy F.V., Weixlbaumer A., Petry S. (2006). Structure of the 70S ribosome complexed with mRNA and tRNA. Science.

[b0185] Shaikh T.R., Gao H., Baxter W.T., Asturias F.J., Boisset N. (2008). SPIDER image processing for single-particle reconstruction of biological macromolecules from electron micrographs. Nature Protocols.

[b0190] Shrum, D.C., Woodruff, B.W., Stagg, S.M., in press. Creating an infrastructure for high-throughput high-resolution cryogenic electron microscopy. Journal of Structural Biology. http://dx.doi.org/10.1016/j.jsb.2012.07.009. 10.1016/j.jsb.2012.07.009 PMC3466351 22842049

[b0195] Sindelar, C.V., Grigorieff, N., in press. Optimal noise reduction in 3D reconstructions of single particles using a volume-normalized filter. Journal of Structural Biology. http://dx.doi.org/10.1016/j.jsb.2012.05.005. 10.1016/j.jsb.2012.05.005 PMC3498508 22613568

[b0200] Sorzano C.O.S., Marabini R., Velazquez-Muriel J., Bilbao-Castro J.R., Scheres S.H.W. (2004). XMIPP: a new generation of an open-source image processing package for electron microscopy. Journal of Structural Biology.

[b0205] Suloway C., Pulokas J., Fellmann D., Cheng A., Guerra F. (2005). Automated molecular microscopy: the new Leginon system. Journal of Structural Biology.

[b0210] Tagare H.D., Barthel A., Sigworth F.J. (2010). An adaptive expectation–maximization algorithm with GPU implementation for electron cryomicroscopy. Journal of Structural Biology.

[b0215] Tang G., Peng L., Baldwin P.R., Mann D.S., Jiang W. (2007). EMAN2: an extensible image processing suite for electron microscopy. Journal of Structural Biology.

[b0220] Wolf M., Garcea R.L., Grigorieff N., Harrison S.C. (2010). Subunit interactions in bovine papillomavirus. Proceedings of the National Academy of Sciences of the United States of America.

[b0225] Wynne S.A., Crowther R.A., Leslie A.G. (1999). The crystal structure of the human hepatitis B virus capsid. Molecular Cell.

[b0230] Yang C., Ji G., Liu H., Zhang K., Liu G. (2012). Cryo-EM structure of a transcribing cypovirus. Proceedings of the National Academy of Sciences of the United States of America.

